# Smart and Rapid Design of Nanophotonic Structures by an Adaptive and Regularized Deep Neural Network

**DOI:** 10.3390/nano12081372

**Published:** 2022-04-16

**Authors:** Renjie Li, Xiaozhe Gu, Yuanwen Shen, Ke Li, Zhen Li, Zhaoyu Zhang

**Affiliations:** 1 Shenzhen Key Laboratory of Semiconductor Lasers, School of Science and Engineering, The Chinese University of Hong Kong, Shenzhen 518172, China; renjieli@link.cuhk.edu.cn (R.L.); yuanwenshen@link.cuhk.edu.cn (Y.S.); keli2@link.cuhk.edu.cn (K.L.); 2 The Future Network of Intelligence Institute (FNii), The Chinese University of Hong Kong, Shenzhen 518172, China; guxiaozhe@cuhk.edu.cn; 3 Department of Computer and Information Engineering, School of Science and Engineering, The Chinese University of Hong Kong, Shenzhen 518172, China

**Keywords:** nanophotonic structures, photonic crystal nanocavities, nanoscale lasers, deep learning, modeling and characterization, neural networks, inverse design

## Abstract

The design of nanophotonic structures based on deep learning is emerging rapidly in the research community. Design methods using Deep Neural Networks (DNN) are outperforming conventional physics-based simulations performed iteratively by human experts. Here, a self-adaptive and regularized DNN based on Convolutional Neural Networks (CNNs) for the smart and fast characterization of nanophotonic structures in high-dimensional design parameter space is presented. This proposed CNN model, named LRS-RCNN, utilizes dynamic learning rate scheduling and L2 regularization techniques to overcome overfitting and speed up training convergence and is shown to surpass the performance of all previous algorithms, with the exception of two metrics where it achieves a comparable level relative to prior works. We applied the model to two challenging types of photonic structures: 2D photonic crystals (e.g., L3 nanocavity) and 1D photonic crystals (e.g., nanobeam) and results show that LRS-RCNN achieves record-high prediction accuracies, strong generalizibility, and substantially faster convergence speed compared to prior works. Although still a proof-of-concept model, the proposed smart LRS-RCNN has been proven to greatly accelerate the design of photonic crystal structures as a state-of-the-art predictor for both *Q*-factor and *V*. It can also be modified and generalized to predict any type of optical properties for designing a wide range of different nanophotonic structures. The complete dataset and code will be released to aid the development of related research endeavors.

## 1. Introduction

Artificial Intelligence (AI) has driven forward the development of countless research disciplines. By applying deep learning on previous data, an AI system can predict future events and make intelligent decisions at a level higher than human beings. At the frontier of deep learning, Deep Neural Networks (DNN) has demonstrated strong robustness and versatility against increasing model depth and data complexity [1,2] and has been widely applied in fields such as facial recognition [3,4] and autonomous driving [5,6]. Excitingly, recent advancement in DNN has given rise to many opportunities for the novel design of nanophotonic and optoelectronic devices, and it has been a central research thrust in the photonics community [7,8,9,10,11,12,13,14,15,16,17,18,19,20,21,22]. This is exemplified by our recent work [20], which modeled 2D photonic crystals using Convolutional Neural Networks (CNNs) and achieved very high prediction accuracies.

DNN has worked well for the following reason: A huge challenge during the design of nanophotonic structures was to correlate the design parameters (e.g., locations, radius, thickness, material selection, etc.) with optical properties (e.g., transmittance, modal volume, power, resonant wavelength, etc.). This correlation was commonly established by iterative physics-based simulations based on the researcher’s prior experience, which is both resource demanding and time consuming. Data-driven methods based on deep learning, on the other hand, can generate complex functions from mega-sized datasets and fit underlying relationships among a large number of complex parameters. Therefore, with enough training data, DNNs can capture this precise design parameter-to-optical property correlation (and its inverse) with more accuracy and little time.

Some very recent works have proposed various DNN models to characterize the relationship between design parameters and optical properties of nanophotonic structures. Ma et al. utilized a CNN model with an encode–decoder paradigm to characterize and design metasurfaces and achieved a mapping from structural pattern to reflectance [12]. Singh et al. applied fully connected (FC) layers to predict the band gap structure from given layer thicknesses of photonic topological designs [13]. Chugh et al. used fully connected layers to model waveguides and studied the relationship between their structural parameters and effective index [15]. Asano et al. applied a CNN to correlate the air hole locations with the *Q*-factor of a 2D photonic crystal and obtained a large *Q*-factor after iterative optimization [10]. Wiecha et al. adopted a CNN to model plasmonic nanostructures and was able to predict their near and far fields from an arbitrarily chosen geometry [18]. Chen et al. modeled photonic metamaterials using an Artifical Neural Network and predicted the absorbance and transmittance from given layer thicknesses [23]. Despite their demonstrated results, all the prior works have failed to address the important issue of overfitting and slow convergence speed that commonly arise in training DNNs. Moreover, most of them only have a small design parameter space and an even smaller optical property space, making their models inadequate for real-world design applications.

For this work, the authors propose a smart deep learning model for designing nanophotonic structures that is based on a self-adaptive and regularized CNN model (named LRS-RCNN by us; meaning of the acronym provided in Section 2.2). As a proof-of-concept, we applied the model to two different types of structures, 2D photonic crystals (e.g., L3 nanocavity) and 1D photonic crystals (e.g., nanobeam), both of which have been extensively studied to enhance their optical properties and are widely adopted in a variety of nanophotonic devices [10,22,24,25,26,27,28,29,30,31,32,33,34,35,36,37,38,39,40,41,42,43]. Nonetheless, the complexity of their periodic structures means that neither one is easy to design. The L3 nanocavity has been previously investigated by us using a CNN to predict only the *Q*-factor [20], and we adopted the same L3 design in this work. LRS-RCNN will function as an accurate predictor for both the *Q*-factor and modal volume *V* and after training, it is demonstrated that record-high prediction accuracies of both optical properties were achieved with fast convergence. Finally, LRS-RCNN was able to generalize extremely well to a fresh validation set previously unobserved by itself and still predicted *Q* and *V* with high fidelity. In summary, this generalized approach has the potential to enable the rapid design of nanoscale lasers and other nanophotonic structures with any set of optical properties.

The main merit and contributions of the proposed model are as follows:

**1**.  *To the best of our knowledge, this is the first time V is modeled by a DNN model as an optical property. V is crucial for reducing device footprints and having tight on-chip integration.*
**2**.  *The employment of CNN empowers the algorithm through its unique advantage on recognizing complex patterns and extracting hidden information from images.*
**3**.  *The use of learning rate scheduling (also known as adaptive learning rate) can effectively smoothen and speed up the convergence of the training process.*
**4**.  *The use of L2 regularization can effectively reduce overfitting and improve the generalizibility of LRS-RCNN.*
**5**.  *It has a high-dimensional design parameter (DA) space with over 160 degrees of freedom. A large DA space is a prerequisite for real-world design problems.*

## 2. Methods

### 2.1. DNN Structure and Architecture

CNN is most powerful when it comes to image-related machine learning tasks [1,2,4,6,44,45]. One could argue that nanophotonic structures such as photonic crystals that seemingly are not related to images can indeed be treated as images, as extensively discussed in our prior work [20]. Thus, one can set up the learning of the optical property predictor as a CNN regression problem. Furthermore, CNN has the ability to compensate for the deficiencies of FC layers when it comes to large design parameter spaces and complex structures [9,20].

**Figure 1:**
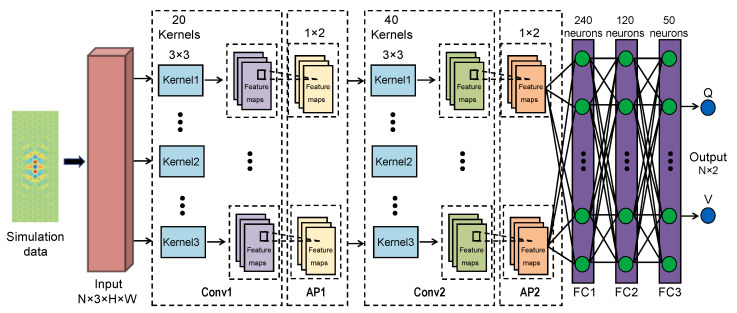
The DNN model (LRS-RCNN) to correlate the input (i.e., design parameters) with output (i.e., optical parameters such as *Q* and *V*) of nanophotonic structures. LRS-RCNN consists of two convolutional layers (Conv1 and Conv2), each accompanied by an averaging pooling (AP) operation, and lastly three FC layers. LRS-RCNN tackles the prediction of optical properties as a deep learning regression problem. The detailed specifications of the network and its hyperparameters are marked on the diagram and/or listed in Table 1.

**Figure 2:**
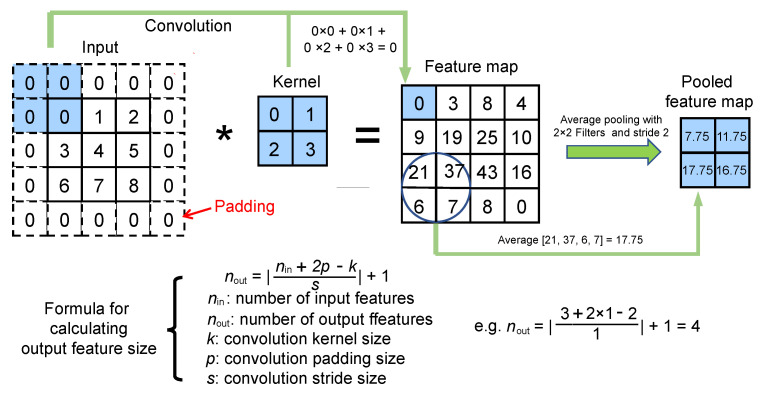
Pictorial illustration of the working principle of convolutional neural networks (CNN) with average pooling. Moreover, the formula for calculating the output feature size of a convolutional operation is also shown.

Figure 1 showcases the CNN model (i.e., LRS-RCNN) built for modeling 2D and 1D photonic crystals in this work. LRS-RCNN consists of two convolutional layers and three FC layers, with the input being a 3-channel N×3×H×W tensor containing user-defined design parameters and the output being an N×2 tensor containing predicted optical properties (*Q* and *V* in our case). *N* represents the batch size, while *H* and *W* correspond to the height and width of the nanophotonic structure, respectively. This specific formalism allows LRS-RCNN to take in a large design parameter space of up to a few hundreds of degrees of freedom (DOF) as input. A full list of the optimized hyperparameters of LRS-RCNN is summarized in Table 1. Rectified linear unit (ReLU, f(x)=max(0,x)) is used as it is both fast and free of vanishing/exploding gradient problems [2,46,47]. Average pooling (AP) can accelerate and stabilize the training of DNN while padding is used to preserve the size of the feature map to avoid information loss at the borders [2,47]. L2 regularization is used in backpropagation to reduce model overfitting [1,2]. Finally, an adaptive learning rate was employed to gain robustness against gradient noise and generate a smoother convergence [1,2,48]. The key working principles of CNN and average pooling are schematically illustrated in Figure 2, where the convolution formula and the feature size formula are also included.

### 2.2. Algorithm Description and Approach

**Figure 3:**
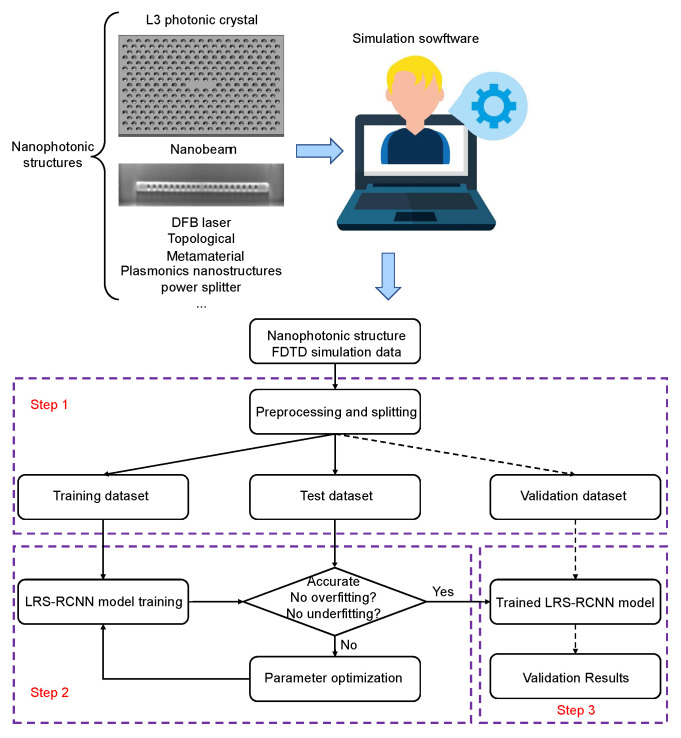
LRS-RCNN algorithm for modeling nanophotonic structures, implemented with a multi-step process: choose the desired structure, randomly fluctuate its design parameters and compute the optical properties in FDTD (pre-training steps), preprocess the dataset (step 1), train the model (step 2), and finally validate the model (step 3). Not shown in the diagram are the adaptive learning rates and L2 regularization, which are both core components of this algorithm.

Figure 3 schematically illustrates the full deep learning algorithm for designing nanophotonic structures by LRS-RCNN. DNNs generally need large datasets for it to effectively learn meaningful experiences and patterns that can aid in the prediction of future events. To generate a training dataset, one chooses the specific structure of interest, randomly fluctuates its design parameters (locations, radii, thicknesses, materials, refractive indices, etc.), and runs Lumerical FDTD [49] simulation to compute the corresponding optical properties (Poyting vector, Q-factor, reflectance, transmittance, resonant frequency, etc.). For our applications, we chose the target photonic crystals and generated 12,750 data samples in FDTD. A simulation of these 12,750 samples was completed in about two weeks; however, the actual time length may vary depending on the type of structures and the computing resources one possesses. We should stress that all data were strictly generated from simulations, and no experimental data/images were produced/used in this work.

After initial data collection, the algorithm follows a 3-step process, as shown in Figure 3: preprocess and split the data, train and optimize the model, and lastly validate the model. Specifically, the dataset is first normalized to have unit standard deviation and then randomly split into three sets—a training set (10,000 data points), a test set (2500 data points), and a validations set (250 data points). These three sets are randomly split up to guarantee that all data features are uniformly distributed to enhance the generalizibility of our model [2]. Normalization was necessary because the input values are on the order of $10^{-9}$ and would have led to vanishing gradients in the training process. During training, one evaluates the training results with the test dataset and closely monitors losses over entire epochs. Care should be taken to ensure the model does not overfit or underfit, both of which are undesirable cases in machine learning [1,2]. After training is complete, as the final step, one should validate the correctness of the learned model by using the validation dataset by checking for signs of any overfitting. It is important to note that the validation data should not have been seen by the model beforehand.

To realize smart and rapid design of nanophotonic structures, we utilized two important techniques that are quintessential parts of the LRS-RCNN algorithm:

1. *Adaptive learning rate* through learning rate scheduling and, thus, the “LRS” in LRS-RCNN. Adaptive learning rate works by dynamically reducing the learning rate when training slows down or a metric hits a plateau and has the power of gaining robustness against gradient noise and inducing a smoother and faster convergence [1,2,48]. While there is a multitude of learning rate schedulers available, Reduce-On-Plateau was selected in this work due to its stable and consistent behavior according to our experiments.
2. *L2 Regularization* and, thus, the “R” after the hyphen in LRS-RCNN. When there is a complex model with a large number of features in the dataset, L2 regularization can be used in backpropagation to address the common overfitting issue and boost generalizibility [1,2]. It works by adding a squared penalty term associated with weight parameters (*W*) to the loss function, as shown in Equation (1), where λ controls how much one would like to penalize large weights. The *Error* term corresponds to *MSE* in Equation (2).
  (1) $$Regularized\;Loss=Error\left(x_{i},x^{*}\right)+\lambda \sum_{i}^{N}W_{i}^{2}$$

Although both techniques have been rarely adopted in the literature, they have brought about tremendous benefits and improvement to the deep learning algorithm as shown later in this article.

Next, the stochastic gradient descent (SGD) optimizer was used to minimize the *loss function*, which is the mean squared error (MSE). Equation (2) calculates the MSE that is defined as the averaged distance between the value $x_{i}$ predicted by LRS-RCNN and the target value $x^{*}$. *x* here could represent any optical properties associated with the chosen structure. $x^{*}$ is also commonly referred to as the “label” in machine learning terminology. In Equations (1) and (2), *N* is the batch size. An accurate predictor with low loss and fast convergence can be realized by optimizing the network hyperparameters listed in Table 1.

(2) $$MSE=\frac{1}{N}\sum_{i}^{N}{\left(x_{i}-x^{*}\right)}^{2}$$

Lastly, the quality of the trained LRS-RCNN will be assessed by a performance metric commonly used in deep learning: the *prediction error* $\epsilon_{pred}$. $\epsilon_{pred}$ represents the relative difference between $x_{i}$ and $x^{*}$ (Equation (3)). In other words, $\epsilon_{pred}=100\%\;-$ prediction accuracy. Both Equations (2) and (3) are part of the closed-loop in Step 2 of Figure 3 for optimizing the model.

(3) $$\epsilon_{pred}=\frac{|x_{i}-x^{*}|}{x^{*}}\times 100\%$$

## 3. Results and Discussion

**Figure 4:**
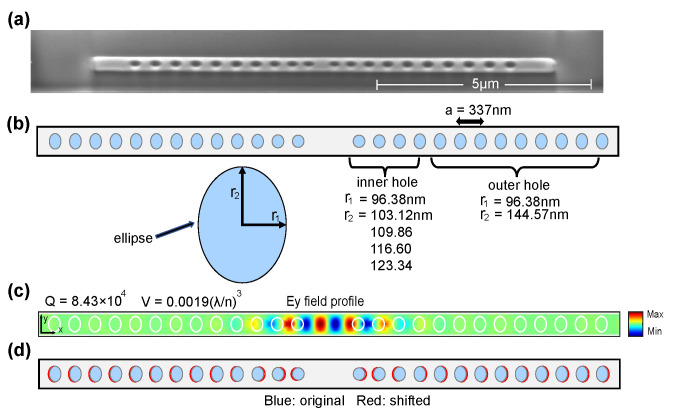
Nanobeam structure. (**a**) SEM image of an actual fabricated nanobeam by us. (**b**,**c**) The initial structure’s design parameters and optical properties, respectively. As shown, holes are elliptically shaped with $r_{1}$ and $r_{2}$ as its two axes. (**d**) A sample in the dataset with randomly shifted holes. Blue: original holes; red: shifted holes.

**Figure 5:**
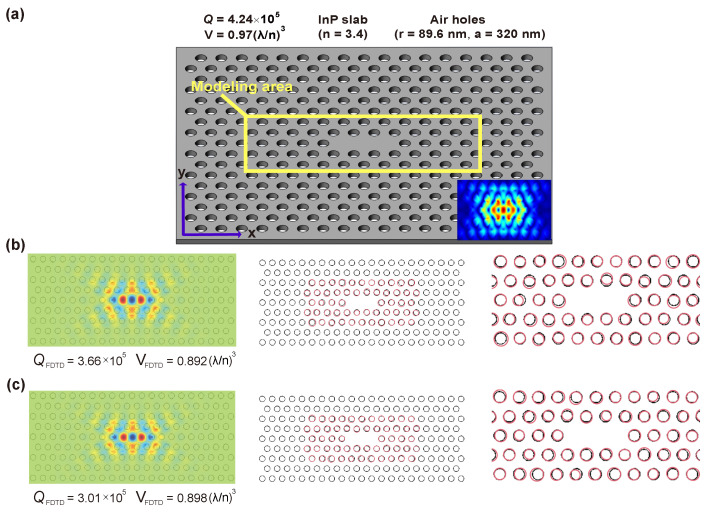
L3 nanocavity structure. (**a**) The initial structure’s design parameters and optical properties. Yellow box is the region where we randomly fluctuate the design parameters. Inset: the $E_{y}$ field of the initial structure. (**b**,**c**) Two samples in the dataset with randomly shifted holes. Black: original holes; red: shifted holes. Far right figures are magnified views of the configuration.

The full-fledged LRS-RCNN model, once properly trained and validated, can be applied as a smart tool to rapidly predict the optical properties of nanophotonic structures. Here, we take a nanobeam and an L3 nanocavity (Figure 4 and Figure 5, respectively) as two examples to demonstrate the power of LRS-RCNN. Figure 4 shows our initial nanobeam design similar to [36,37], where Figure 4a is the SEM image of an actual InP nanobeam fabricated by us. Figure 4b illustrates the original design parameters including semi-minor axis, semi-major axis, lattic constant *a*, and number of holes, while Figure 4c showcases the corresponding $E_{y}$ filed profile, *Q*-factor, and modal volume *V*. Figure 4d illustrates how the holes have been randomly shifted relative to Figure 4b to generate the dataset as laid out in Section 2.2. The details of the dataset generated are summarized in Table 2. Similarly to nanobeams, Figure 5 shows our initial L3 nanocavity design where Figure 5a contains the original design parameters and optical properties and Figure 5b,c are two samples in the dataset with randomly shifted holes. The L3 nanocavity dataset is also summarized in Table 2. Since nanobeam has 13 holes on each side (symmetrically shifted) and each hole has three design parameters, there is a total of 39 DOFs; by the same token, the L3 nanocavity is calculated to have 162 DOFs (the complete dataset and its detailed description can be found at [50]).

### 3.1. Nanobeam

**Figure 6:**
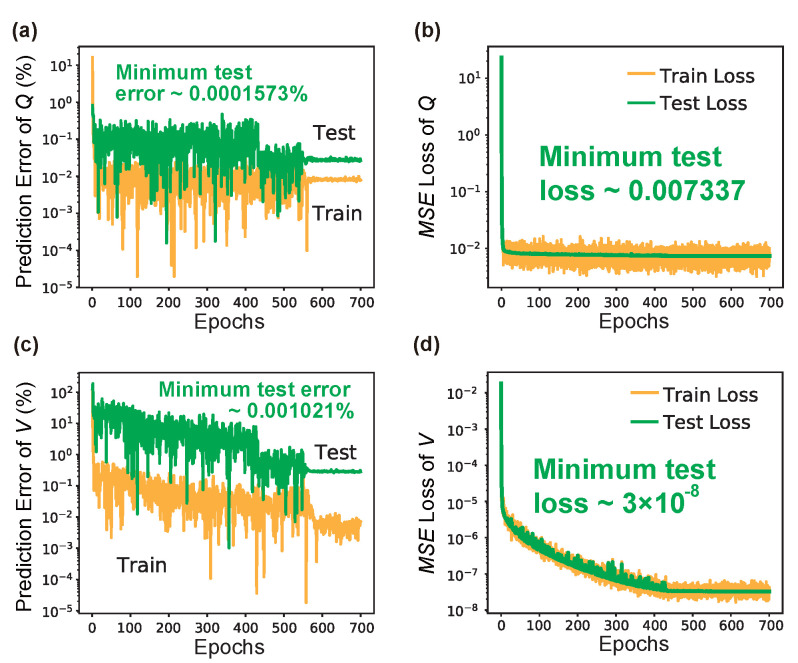
Learning curves of LRS-RCNN when trained to characterize nanobeam’s *Q* and *V*. Top panel contains results for *Q*: (**a**) prediction error $\epsilon_{pred}$ vs. Epochs curve; (**b**) MSE vs. Epochs curve, for both training (yellow) and test (green) datasets. Min $\epsilon_{pred}$ and MSE using the test dataset are also labeled on the figures, respectively. Bottom panel are the same results for *V* (**c**,**d**).

Figure 6 shows the learning results of LRS-RCNN when trained to model the nanobeam. Figure 6a,b are the convergence curves of $\epsilon_{pred}$ and MSE over the entire epochs for *Q*, respectively, while Figure 6c,d are those for *V*. Key performance metrics using the test dataset have been extracted from Figure 6 and summarized in Table 3 for comparison to prior works, where bold-faced numbers indicate the best metrics among all listed works. As seen in Table 3, in the case of the nanobeam, LRS-RCNN yielded dominant performance and contributed three best metrics—min $\epsilon_{pred}$ for *Q*, min MSE, and $\bar{MSE}$ for *V*—that are multiple orders of magnitude smaller than existing literature data [10,13,14,15,18,22,23,51]. Furthermore, some of the other metrics, such as prediction time for a single structure and time speedup relative to conventional FDTD simulation (over six orders of magnitude), were also placed high up on the leaderboard. These results have demonstrated LRS-RCNN’s state-of-the-art capacity for the smart and rapid design of the nanobeam.

**Figure 7:**
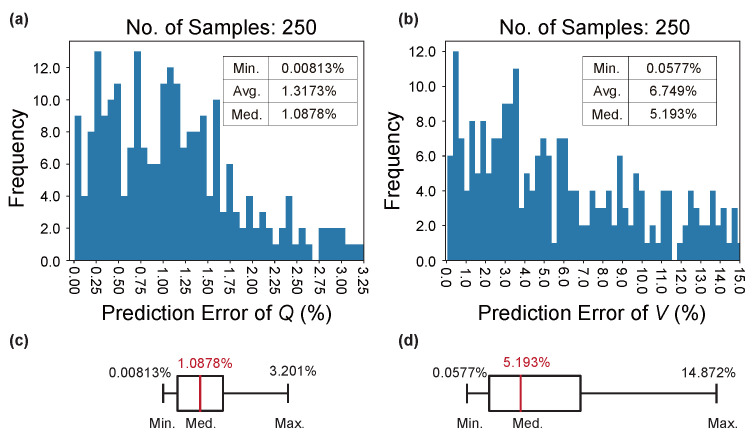
Validation results of the trained LRS-RCNN for nanobeam using the validation dataset. Plotted is the histogram of $\epsilon_{pred}$ for *Q* in (**a**) and *V* in (**b**). The boxplot in (**c**) is extracted from data in (**a**), and (**d**) from (**b**). Inset data: minimum, average, and median $\epsilon_{pred}$ values in the histograms.

Next, Figure 7 exhibits the validation results of the trained LRS-RCNN using the validation dataset, where the prediction error $\epsilon_{pred}$ for both the *Q* and *V* are plotted. This step is necessary for verifying the model’s ability to generalize unknown design parameters and for checking for any presence of overfitting. As shown in Figure 7a,c, statistically speaking, an average $\epsilon_{pred}$ of 1.317% and a median $\epsilon_{pred}$ of 1.088% warrant a prediction accuracy for *Q* close to 99% (the highest so far in the literature). Similarly, according to Figure 7b,d, the prediction accuracy for *V* is approaching 95%, which is still considered highly accurate for DNNs. Although the highest $\epsilon_{pred}$ for *V* is close to 15%, it can be ignored as outliers as shown in the boxplot Figure 7d. These validation results guarantee that LRS-RCNN has attained excellent generalizibility as a predictor for both optical properties.

Lastly, it should be noted that since this is the first time *V* has been included in any deep learning based modeling of photonic crystals in the literature, the authors could only compare the training results of *V* against their own results of *Q* during the production of this work. The authors hope what is demonstrated here could be used as a benchmark for future work.

**Table 3:** Results of training LRS-RCNN with nanobeam and L3 nanocavity. Tabulated are the performance metrics of the predictor at the test/validation phase and its comparison against prior works. Data partially extracted from Figure 6 and Figure 8. Overlined parameters, such as $\bar{\epsilon_{pred}}$ and $\bar{MSE}$, indicate converged values. Bold-faced numbers are best metrics. Prediction time and speedup vs. FDTD, both calculated by averaging the time measured with samples in the validation dataset, refer to the time for characterizing a single structure by LRS-RCNN. N/A means no data available from the cited work.

		min $\epsilon_{pred}$	$\bar{\epsilon_{pred}}$	min MSE	$\bar{\mathrm{MSE}}$	Epochs	Training Time	Prediction Time	Speedup (vs. FDTD)
**Nanobeam**	*Q*	**0.000157%**	0.0148%	0.00734	0.00735	700	9 min	$7\times 10^{-5}$ s	$5.1\times 10^{6}$
	*V*	0.00102%	0.141%	$3.00\times 10^{-8}$	$3.16\times 10^{-8}$				
**L3**	*Q*	0.00105%	**0.0112%**	0.000360	0.000365	300	5 min	$9\times 10^{-5}$ s	$3\times 10^{6}$
	*V*	0.00317%	0.0158%	0.00252	0.00282				
Gou et al. [22]		0.540%	N/A	0.000300	N/A	1000	143 s	$3.6\times 10^{-5}$ s	$1.2\times 10^{8}$
Christensen et al. [14]		0.470%	0.530%	N/A	0.006	400	3 min	0.02 s	N/A
Asano et al. [10]		16.0%	19.0%	N/A	N/A	$2\times 10^{5}$	N/A	N/A	N/A
Chugh et al. [15]		1.00%	N/A	N/A	0.00808	4000	N/A	N/A	N/A
Singh et al. [13]		N/A	N/A	0.00810	0.014	500	45 min	N/A	N/A
Chen et al. [23]		0.700%	5.00%	N/A	N/A	110	N/A	N/A	N/A
Wiecha et al. [18]		N/A	5.78%	N/A	N/A	**100**	200 min	$3\times 10^{-3}$ s	$10^{5}$
Tahersima et al. [51]		N/A	N/A	N/A	0.130	10,000	22 min	N/A	N/A

### 3.2. L3 Nanocavity

**Figure 8:**
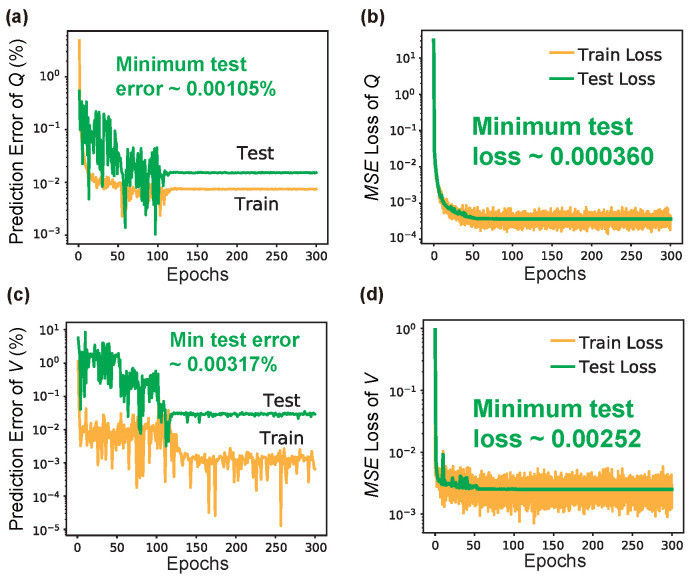
Learning curves of LRS-RCNN when trained to characterize L3 nanocavity’s *Q* and *V*. Top panel contains results for *Q*: (**a**) prediction error $\epsilon_{pred}$ vs. Epochs curve; (**b**) MSE vs. Epochs curve, for both training (yellow) and test (green) datasets. Min $\epsilon_{pred}$ and MSE using the test dataset are also labeled on the figures, respectively. Bottom panel are the same results for *V* (**c**,**d**).

In the same fashion as the nanobeam, Figure 8 shows the learning results of LRS-RCNN when trained to model the L3 nanocavity, the metrics data of which have also been summarized in Table 3. Although LRS-RCNN only contributed one best metric (i.e., $\bar{\epsilon_{pred}}$ for *Q*), its other metrics are all ranked high on the leaderboard and are comparable to the best metrics reported for the nanobeam. For instance, the min $\epsilon_{pred}$, $\bar{MSE}$, and prediction times are better than and/or comparable to those reported in prior works [10,13,14,15,18,22,23,51] by a large margin. Moreover, the Epochs until convergence, which include 300, leads to super fast convergence and largely reduced training time. Therefore, we have once again demonstrated the power of LRS-RCNN for the smart and rapid design of L3 nanocavities.

**Figure 9:**
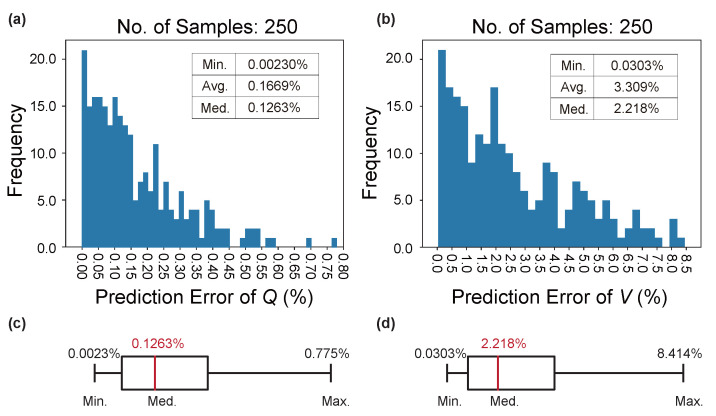
Validation results of the trained LRS-RCNN for L3 nanocavity using the validation dataset. Plotted is the histogram of $\epsilon_{pred}$ for *Q* in (**a**) and *V* in (**b**). The boxplot in (**c**) is extracted from data in (**a**), and (**d**) from (**b**). Inset data: minimum, average, and median $\epsilon_{pred}$ values in the histograms.

As for validation, one can see that in Figure 9a, an average $\epsilon_{pred}$ of 0.167% and a median $\epsilon_{pred}$ of 0.126% warrant a prediction accuracy for *Q* close to 99.9% (the highest so far in the literature). Similarly, according to Figure 9b, the prediction accuracy for *V* is approaching 97%, which is generally regarded as highly accurate for DNNs. Although the max $\epsilon_{pred}$ for *V* is as high as 8.4%, it can be ignored as outliers as illustrated in the boxplot of Figure 9d. The validation results here can reinforce the fact that LRS-RCNN is a well generalizible predictor for unseen optical properties.

### 3.3. On the Importance of Adaptiveness and Regularization

**Figure 10:**
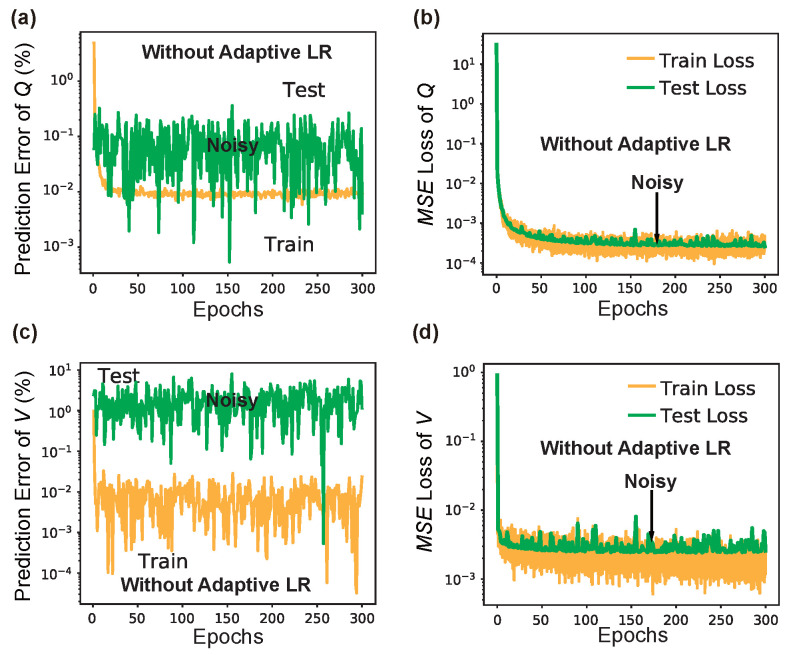
Results of training LRS-RCNN for L3 nanocavity when *no* LRS is used. The figure has the same order as Figure 8. One can observe the substantially noisier convergence curves in (**a**–**d**) in this case compared to before.

**Figure 11:**
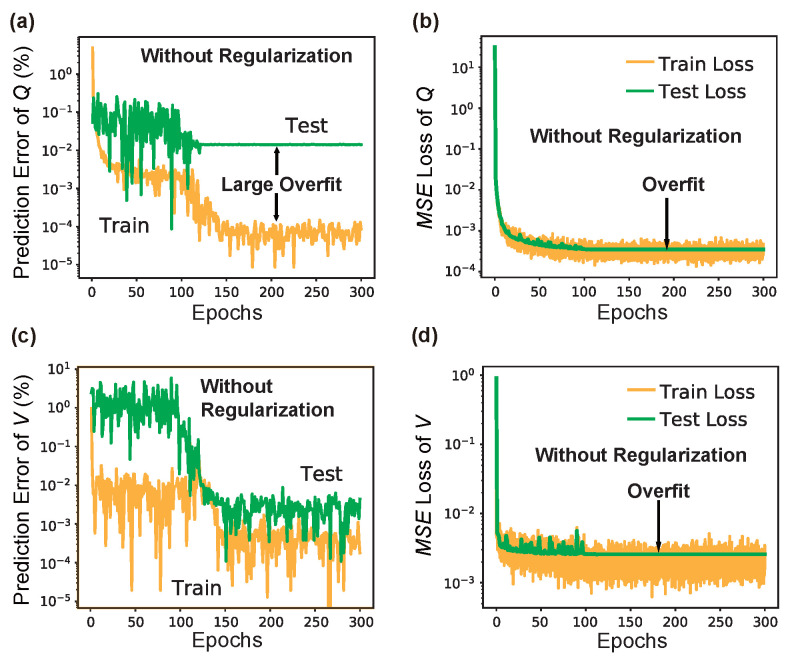
Results of training LRS-RCNN for L3 when *no* L2 regularization is used. The figure has the same order as Figure 8. It is observed that large overfittings are present in (**a**–**d**) in this case.

The significant impacts learning rate scheduling (LRS) and L2 regularization have on the deep learning model is discussed here. For simplicity, we only conducted experiments with L3 nanocavity, and similar results should easily extend to nanobeam and other structures. For the comparative experiment, the learning curves of LRS-RCNN *without* using LRS and L2 regularization are compared to those shown previously in Figure 8. It should be noted that the exact same hyperparameters and dataset are used here for training. Figure 10 shows the results without using LRS, where it can be observed that the learning curves are much more noisier than those in Figure 8 and they even failed to converge within the initial 300 epochs. This means a much slower convergence speed. Next, in Figure 11, one can observe the existence of large overfittings in all of them, but (c) when the L2 regularization is not used, which means poor generalizibility of the trained model. Therefore, we can conclude that both LRS (i.e., adaptive learning rate) and L2 regularization are crucial for the realization of smart and rapid designs.

## 4. Conclusions

In conclusion, this work has proposed and successfully demonstrated a self-adaptive and regularized CNN model for designing nanophotonic structures. The smart LRS-RCNN with dynamic learning rate scheduling and L2 regularization allows one to rapidly predict optical properties with high confidence from a large design parameter space. Specifically, when tested on two different photonic crystal structures, all major performance metrics improved by several orders of magnitude compared to previously reported values and, thus, proved that LRS-RCNN reached a state-of-the-art capacity. Using a validation set not previously seen, LRS-RCNN was still able to predict *Q* and *V* with an accuracy up to 99.9% and 97%, respectively. This demonstrates the strong generalizibility of the trained model that allows for the prediction of optical properties from an arbitrary set of design parameters or even arbitrary nanophotonic structures. In addition, since this is the first time *V* has been characterized by a deep learning model, we hope our work can serve as a benchmark for assessing related works in the future. Lastly and more broadly, this generalized accurate predictor potentially paves the way for the rapid design of a series of optoelectronic and photonic integrated devices [32,52,53,54,55,56,57,58,59,60,61] with an extremely high performance caliber.

A limitation of this work might be a lack of experimental data that back the numerical findings. However, given Lumerical FDTD’s proven strong fidelity relative to experimental data, we can say with confidence that the results in Figure 6, Figure 7, Figure 8 and Figure 9 and Table 3 are highly accurate and reliable. Nonetheless, using experiments to support calculations is still an important component of scientific research and worth our attention going forward. Efforts could further develop our architecture to predict more optical properties, including resonance frequency, full width at half maximum and lasing threshold, etc., all of which are key attributes of lasers based on photonic crystals. This would likely require the adoption of a more powerful DNN, and the latest development in vision transformers [62] and attention nets [63] are promising candidates to choose from. In addition, data collection so far takes up to two weeks to complete due to the large computational complexity of FDTD simulations. To resolve this, we would turn to some open-source light-weight simulation packages such as MPB and MEEP that would greatly reduce time consumption at the cost of losing certain degree of accuracy. Another benefit of this alternative approach would be the ability to collect more data. Furthermore, we could experiment our algorithm on a more diverse pool of structures, such as plasmonic structures, metamaterials, and DFB lasers, all of which are important and interesting paths to pursue. Lastly, instead of relying on aimlessly cascading DNNs or gradient-based optimization algorithms, we could employ the latest reinforcement learning models [64] to inverse design and optimize nanophotonic structures on a large scale.

## Figures and Tables

**Table 1:** Hyperparameters of LRS-RCNN.

Hyperparameter	Value	Hyperparameter	Value
Conv1	20 3 × 3 kernels + 1 × 2 AP	FC1	240 neurons
Conv2	40 3 × 3 kernels + 1 × 2 AP	FC2	120 neurons
Paddings	1	FC3	50 neurons
Activation function	ReLU	No. of Epochs	700
Training batch size	64	Test batch size	100
Optimizer	SGD	Initial learning rate	0.01
Momentum	0.5	L2 regularization λ	0.001
Nanobeam’s (H, W)	(1, 13)	Learning rate scheduler	ReduceOnPlateau
L3’s (H, W)	(5, 12)	Loss function	MSE

**Table 2:** Specifications of the dataset, generated according to Section 2.2, for training LRS-RCNN (DA = design parameter, OP = optical property, and DOF = degree of freedom). Left panel: nanobeam; right panel: L3 nanocavity. DAs such as “*x* locations” and “*r*” refer to the x coordinate and radius of each individual air hole, respectively. Corresponding OPs are computed by FDTD simulation.

Nanobeam DAs	Nanobeam OPs	L3 DAs	L3 OPs
$r_{1}$	*Q*-factor	*x* locations	*Q*-factor
$r_{2}$	Modal volume *V*	*y* locations	Modal volume *V*
*x* locations		*r*	
DOF=13×3=39		DOF=54×3=162	

## Data Availability

The data and code presented in this study are openly available in Github at https://github.com/Arcadianlee/Deep-Learning-Design-Photonic-Crystals.git (accessed on: 10 November 2021), reference number [50]. Full deep learning code and simulation files used in the production of this work are available from R.L. upon reasonable request at the current stage and will be made publicly available in the future following the completion of this project.
